# Sequence and epigenetic landscapes of active and silent nucleolus organizer regions in *Arabidopsis*

**DOI:** 10.1126/sciadv.adj4509

**Published:** 2023-11-01

**Authors:** Dalen Fultz, Anastasia McKinlay, Ramya Enganti, Craig S. Pikaard

**Affiliations:** ^1^ Howard Hughes Medical Institute, Indiana University, Bloomington, IN, USA.; ^2^ Department of Biology, Indiana University, Bloomington, IN, USA.; ^3^ Department of Molecular and Cellular Biochemistry, Indiana University, Bloomington, IN, USA.

## Abstract

*Arabidopsis thaliana* has two ribosomal RNA (rRNA) gene loci, nucleolus organizer regions *NOR2* and *NOR4*, whose complete sequences are missing in current genome assemblies. Ultralong DNA sequences assembled using an unconventional approach yielded ~5.5- and 3.9-Mbp sequences for *NOR2* and *NOR4* in the reference strain, Col-0. The distinct rRNA gene subtype compositions of the NORs enabled the positional mapping of their active and inactive regions, using RNA sequencing to identify subtype-specific transcripts and DNA sequencing to identify subtypes associated with flow-sorted nucleoli. Comparisons of wild-type and silencing-defective plants revealed that most rRNA gene activity occurs in the central region of *NOR4*, whereas most, but not all, genes of *NOR2* are epigenetically silenced. Intervals of low CG and CHG methylation overlap regions where gene activity and gene subtype homogenization are high. Collectively, the data reveal the genetic and epigenetic landscapes underlying nucleolar dominance (differential NOR activity) and implicate transcription as a driver of rRNA gene concerted evolution.

## Teaser

Sequencing and activity mapping of *Arabidopsis thaliana* NORs reveals the epigenetic parameters of their alternative on/off states.

## INTRODUCTION

In eukaryotes, ribosomal RNA (rRNA) genes transcribed by DNA-dependent RNA polymerase I are repeated in long tandem arrays, typically spanning millions of base pairs (*1*, *2*). These chromosomal loci are known as nucleolus organizer regions (NORs) because the nucleolus, the most prominent substructure of the nucleus, forms around active rRNA genes as their transcripts are processed into 18*S*, 25*S* to 28*S*, and 5.8*S* catalytic rRNAs (*3*, *4*). These rRNAs assemble with ~80 ribosomal proteins and 5*S* rRNA to form ribosomes, the protein-synthesizing machines of cells (*5*–*7*).

rRNA gene repeats within a species are extremely similar in sequence complexity yet can be differentially expressed, accounting for the epigenetic phenomenon known as nucleolar dominance (*8*–*10*). This phenomenon was initially discovered in genetic hybrids and described the preferential activity of NORs inherited from one progenitor. However, nucleolar dominance is not unique to hybrids, also occurring in pure species (nonhybrids) to regulate the number of active rRNA gene repeats at different times in development (*9*, *11*, *12*).

In *Arabidopsis thaliana* strains Col-0 and Ler, genetic and physical mapping studies indicated that an estimated 375 to 400 rRNA genes are present within each of two NORs, *NOR2* and *NOR4*, located at the beginnings of chromosomes 2 and 4 and capped by telomere repeats (*13*–*15*). Variability at the 3′ ends of the genes led to the finding that one subclass (VAR1 genes), accounting for approximately 40 to 50% of the total rRNA gene population in Col-0, is silenced during early postembryonic development (*16*, *17*) in a process involving DNA cytosine methylation and histone deacetylation (*18*, *19*). By contrast, genes of the VAR2, VAR3, and VAR4 subclasses were (at least partially) expressed. Genetic segregation analyses of the 3′ end polymorphisms and a small set of additional differentially expressed polymorphic markers revealed that genes bearing nonexpressed polymorphisms mapped to *NOR2,* whereas active genes mapped to *NOR4* (*20*, *21*). Subsequently, a Col-0 line was identified in which a large portion of *NOR4* was replaced by sequences of *NOR2.* In this line, the transposed *NOR2* genes escaped silencing (*22*). Collectively, these findings indicated that rRNA gene activity is not dictated by individual gene sequences but, instead, somehow depends on NOR affiliation, highlighting a need to better understand the functional organizations of *A. thaliana* NORs and eukaryotic NORs in general (*23*, *24*). In the case of *A. thaliana*, complete NOR sequences have proven difficult to assemble, with the longest contigs (overlapping sequencing reads representing a contiguous region) before our study being limited to the edges of the NORs and consisting of fewer than 50 rRNA genes (*25*–*27*). We thus set out to obtain end-to-end sequences of *NOR2* and *NOR4* to define their gene subtype compositions, regions of gene activity, and regions where chromatin-mediated silencing occurs, thereby revealing the full scope of nucleolar dominance as it occurs in the context of complete NORs.

## RESULTS

### Variable-length elements define at least 74 rRNA gene subtypes

**Fig. 1.**
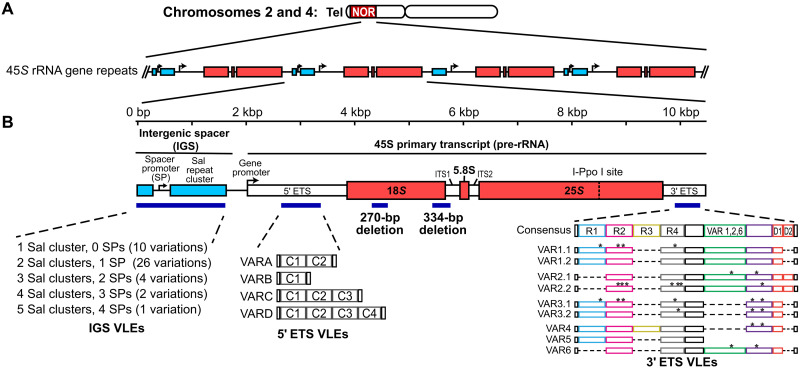
Organization of the *Arabidopsis thaliana* nucleolus organizer regions (NORs). (**A**) The NORs abut the telomeres on the short arms of chromosomes 2 and 4 and consist of tandemly repeated copies of 45*S* ribosomal RNA (rRNA) genes. (**B**) Diagram of a single rRNA gene repeat, showing the locations of VLEs. “C” repeats in the 5′ external transcribed spacer (5′ETS) are 310 bp in length. Colors used to differentiate subrepeats of the 3′ETS region are used to help visually compare their permutations among the different variable-length elements (VLEs). Asterisks in the 3′ETS “R” repeats and elsewhere denote single-nucleotide polymorphisms (SNPs) relative to a consensus reference sequence.

At *NOR2* and *NOR4*, rRNA genes are repeated head to tail and oriented such that transcription occurs toward the centromere (Fig. 1A) (*14*, *27*). Each transcription unit is separated by an intergenic spacer (IGS) that includes the gene promoter, 0 to 4 spacer promoters that share homology with core sequences of the gene promoter and can program low-level transcription by RNA polymerase I (*28*), and variable numbers of short repeats bearing Sal I restriction endonuclease sites (*29*). Sequence elements within the transcribed region include a 5′ external transcribed spacer (5′ETS), 18*S*, 5.8*S*, and 25*S* catalytic rRNAs, two internal transcribed sequences, ITS1 and ITS2, and a 3′ETS. Gene promoter, rRNA, and ITS sequences are nearly identical among rRNA gene repeats, but deletions within the 18*S* region occur in a small number of genes (Fig. 1B) (*27*). Notably, these partially deleted 18*S* rRNAs are not detected in assembled ribosomes (*27*). Substantial variation occurs within IGS and ETS regions due to variable numbers of Sal repeats, spacer promoters, 5′ETS repeats, or 3′ETS repeats, making each of these elements variable in length (Fig. 1B). Using Pacific Bioscience SMRT sequencing of ~10–kilobase pair (kbp) rRNA gene units cut from genomic DNA (gDNA) by I-Ppo I (fig. S1) and subsequent Oxford Nanopore sequencing of uncut gDNA (see below), we expanded upon the variation known from other studies (*27*, *29*, *30*), doubling the number of variable-length elements (VLEs). The 59 VLEs summarized in Fig. 1B (58 plus the reference sequence) occur in 74 permutations, thus defining a minimum of 74 rRNA gene subtypes (fig. S2A).

### Multigene VLE patterns allow NOR2 and NOR4 assembly from ultralong DNA sequences

Sequence conservation among rRNA genes precludes their discrimination using short-read DNA sequencing, making NORs notoriously difficult loci to assemble. However, we hypothesized that rare VLE patterns spanning multiple genes might provide landmarks for NOR assembly. Oxford Nanopore Technology (ONT) sequencing of *A. thaliana* gDNA yielded reads that averaged ~40 to 50 kbp but included numerous reads longer than 200 kbp (table S1). Whole-genome assembly using the long ONT reads recapitulated the TAIR10 (The Arabidopsis Information Resource, release 10) genome assembly (*31*) for the *A. thaliana* reference strain, Col-0 (fig. S3), confirming comprehensive genome coverage within the read set. However, neither NOR was substantially assembled, as is the case for the TAIR10 reference genome and more recent assemblies based on long-read sequencing technologies (*25*–*27*).

**Fig. 2.**
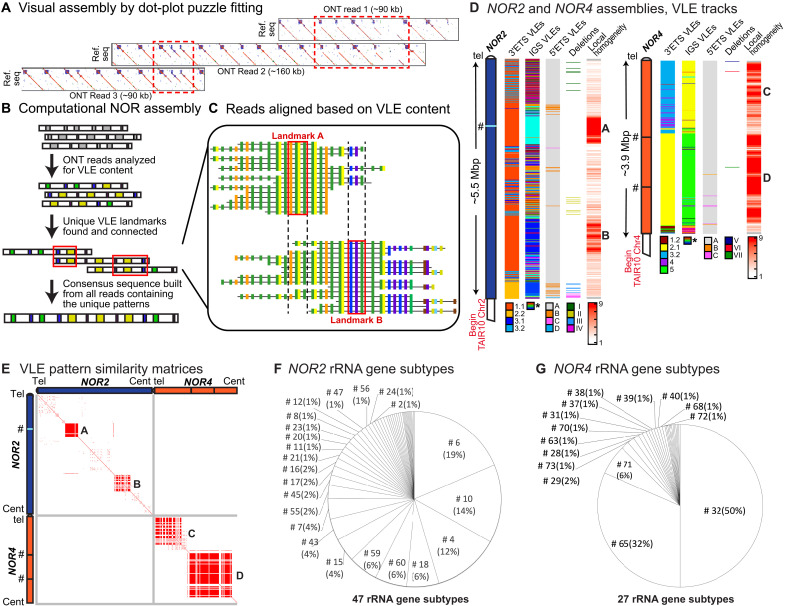
Assembly of *NOR2* and *NOR4*. (**A**) Example of an assembly of overlapping dot plots. (**B**) Schematic of the computational assembly pipeline. (**C**) Diagram depicting aligned reads at two adjacent variable-length element (VLE) landmarks. Within each read, colored boxes represent different VLEs. (**D**) Graphical summary of the nucleolus organizer region (NOR) assemblies, with tracks for distinct VLEs, partial gene deletions, and local homogeneity (number of identical gene subtypes within a nine-gene sliding window). Color codes are provided below each track, except for intergenic spacer (IGS) VLEs, which have too many types and associated colors (43) to provide a meaningful color code. The # symbols indicate positions in the assembly where contigs were joined manually because the same gene subtype is repeated for such long distances that they were not spanned by individual Oxford Nanopore Technology (ONT) reads. (**E**) Similarity matrices comparing VLE content within and between NORs. Each red point represents a perfect match of VLEs, within a three-gene window, in the sequences being compared. (**F** and **G**). Pie charts representing the relative abundance of gene subtypes within *NOR2* or *NOR4*. See fig. S2 for subtype details. rRNA, ribosomal RNA.

To fully assemble the NORs, we used two approaches in parallel. In one approach, ONT reads containing rRNA gene sequences were compared to a single rRNA gene reference sequence, namely, the sequence of gene subtype #10 (see data file S1 and fig. S2) to generate two-dimensional similarity matrices (dot plots). Individual dot plots displaying distinct and rare patterns were then manually assembled, like puzzle pieces, yielding longer contigs, as illustrated in Fig. 2A. The second approach involved a computational NOR assembly pipeline (Fig. 2B). The pipeline first determined the order of VLEs within long (>85 kbp) rRNA gene–containing ONT reads that collectively provided ~100× coverage [based on an estimate of ~750 to 800 rRNA genes per haploid genome (*15*); see Materials and Methods]. We then identified sets of VLEs predicted to be unique based on their frequency within the read set (data file S2) and used them as landmarks for aligning and grouping overlapping ONT reads, ultimately connecting the landmarks (see Fig. 2C and data files S3 to S7) to yield full-length assemblies of ~5.5 mega–base pairs (Mbp) for *NOR2* and 3.9 Mbp for *NOR4* (Fig. 2D). Multiple sequence alignment of overlapping reads enabled consensus sequence polishing for both NORs (see Materials and Methods), with comparisons to 18*S* and 25*S* rRNA sequences, which are essentially invariant (*21*), indicating that the final *NOR2* and *NOR4* sequences have a base pair accuracy of ~99.85% (data file S8). The polished, fully assembled *NOR2* and *NOR4* sequences are available at GenBank as accessions OR453402 (https://ncbi.nlm.nih.gov/nuccore/OR453402) for *NOR2* and OR453401 (https://ncbi.nlm.nih.gov/nuccore/OR453401) for *NOR4*.

We quality checked the *NOR2* and *NOR4* assemblies in three ways. First, we compared the frequency of VLE patterns in ONT reads versus the NOR assemblies, obtaining a correlation value of *R*^2^ = 0.985 (data file S9.) This indicates that the assemblies reflect the VLE patterns in the raw data. Second, ONT reads of computationally assembled contigs were visually examined using the dot-plot puzzle-fitting approach. Third, we used custom-designed CRISPR guide RNAs complementary to alternative 3′ETS VLEs to catalyze Cas9 cutting of gDNA. We then separated the DNA fragments by contour-clamped homogeneous electric field (CHEF) electrophoresis and performed Southern blotting with a rRNA probe (fig. S4). The Cas9 digestion products were consistent with those predicted from the NOR assemblies.

*NOR2* has 518 rRNA gene repeats, based on the count of 25*S* rRNA sequences, whereas *NOR4* has 376 genes. Individual rRNA gene units average ~10 kb but vary in size from 7812 to 12,205 bp, as measured by the distance between the I-*Ppo* I endonuclease cutting sites that occur once per gene (see Fig. 1). However, it is important to note that the shortest units are those with substantial internal deletions expected to render them nonfunctional.

Figure 2D shows the NOR organizations with respect to 3′ETS, IGS and 5′ETS VLEs, or internal deletions, each displayed as a vertical track. Another track depicts the number of times any single rRNA gene subtype is repeated within a sliding window spanning nine genes (~90 kbp), providing a measure of local subtype homogeneity. VLE distribution patterns are unique to each NOR, as illustrated in similarity matrices comparing VLE features within and between the NORs (Fig. 2E).

### NOR2 and NOR4 have unique and nonoverlapping rRNA gene subtype compositions

**Fig. 3.**
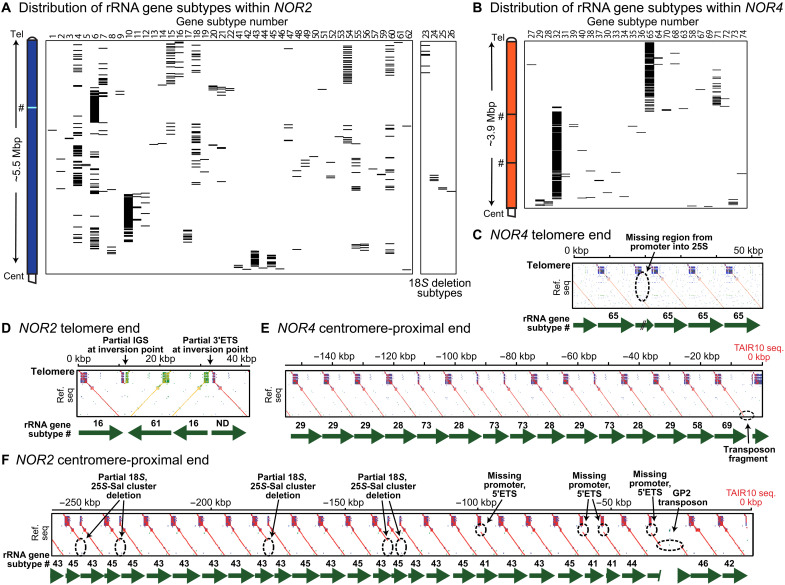
Structural features of *NOR2* and *NOR4*. (**A** and **B**) Positions of individual gene subtypes throughout *NOR2* and *NOR4*. The group separated at the right of (A) are subtypes with deletions in 18*S* ribosomal RNA (rRNA) sequences. (**C** to **F**) Similarity matrices for the telomere and centromere-proximal ends of *NOR2* and *NOR4*. Distinctive features are highlighted with dashed circles. Numbering is relative to the telomere-rRNA gene junction in (C) and (D) and relative to the start of the TAIR10 chromosome 2 and 4 assemblies for (E) and (F). # symbols indicate positions in the assembly where contigs were joined manually, as described for Fig. 2D.

The NOR sequence assemblies revealed that there are individual VLEs that are shared by genes at *NOR2* and *NOR4*, but none of the 74 rRNA gene subtypes are common to both NORs (figs. S2B and S5). *NOR2* is composed of 47 rRNA gene subtypes [Figs. 2 (F and G) and 3 (A and B)], mostly VAR1.1 class genes (subtypes 1 to 26 in Fig. 3A and fig. S2A) interspersed with VAR3 genes of both the 3.1 (subtypes 47 to 53) and 3.2 subclasses (subtypes 54 to 70). Two regions of high local subtype homogeneity account for ~25% of *NOR2* (Fig. 2, D and E). Homogeneous region A is enriched for gene subtype 6 (Fig. 3A), the most abundant *NOR2* subtype, comprising 19% of the NOR (Fig. 2F). Region B is enriched for subtype 10, the second most abundant *NOR2* subtype (14%; Figs. 2F and 3A).

*NOR4* is composed of 27 rRNA gene subtypes (Fig. 2G and fig. S2B), with three accounting for 88% of the NOR (Fig. 2G) and clustering within homogeneous regions C and D (Fig. 2, D and E). Region C is composed of VAR3.2 class genes, primarily subtype 65 genes that account for 32% of *NOR4* (Figs. 2G and 3B), interspersed with VAR4 class genes, primarily subtype 71 genes comprising 6% of the NOR (Figs. 2G and 3B). Region D consists primarily of VAR2.1 class genes, specifically subtype 32, which comprises 50% of *NOR4* (Figs. 2G and 3B).

At two positions within *NOR4*, marked by “#” symbols in Fig. 2D (and subsequent figures), the contigs on either side (data files S5 to S7) end in long stretches of subtype 32 repeats that were joined manually to form a gapless assembly. Because no ONT reads were long enough to span the entirety of these regions, with unique landmarks at each end, the precise number of subtype 32 repeats is unclear. Comparison of subtype 32 abundance in raw sequencing reads versus the *NOR4* assembly suggests that ~29 copies of subtype 32 may be unaccounted for in the assembly (data file S9). If so, the additional genes would be present at one or both locations indicated by the # symbols, potentially making *NOR4* slightly longer than the assembled sequence. Likewise, at one position in *NOR2* (also denoted by #), the contigs for the telomere-proximal and centromere-proximal ends of the NOR (data files S3 and S4) join within a long uninterrupted array of subtype 6 genes in homogeneous region A. These contigs were also joined manually to form a gapless assembly, but in this case, the number of subtype 6 genes in the assembly is in good agreement with the total estimated number (data file S9).

The telomere and centromere-proximal ends of *NOR2* and *NOR4* have distinct structural features (Fig. 3, C to F). At the telomeric end of *NOR4*, the third gene has an internal deletion of several kilobase pairs (Fig. 3C), one of only four structural anomalies within the entirety of *NOR4*. The other anomalies are two genes with 25*S* deletions (Fig. 2D, “Deletions” track) and one gene, near the centromere-proximal end, disrupted by a partial GP2 (Gypsy family) transposon (Fig. 3E). By contrast, *NOR2* has numerous genes with structural anomalies. At the telomere-proximal end, an inversion of ~20 kbp has occurred (Fig. 3D). At the centromere-proximal end, more than one-third of the genes in the final 250 kbp have internal deletions eliminating their gene promoter regions, and a GP2 transposon interrupts the third gene from the end (Fig. 3F). In central regions of *NOR2*, the two types of 18*S* deletions (*27*) occur in several nearby copies. In total, *NOR2* has ~20 genes whose internal deletions would likely render them nonfunctional (see Fig. 2D, Deletions track).

### Polymorphisms detected by RNA-seq allow the physical mapping of active and silenced rRNA genes

**Fig. 4.**
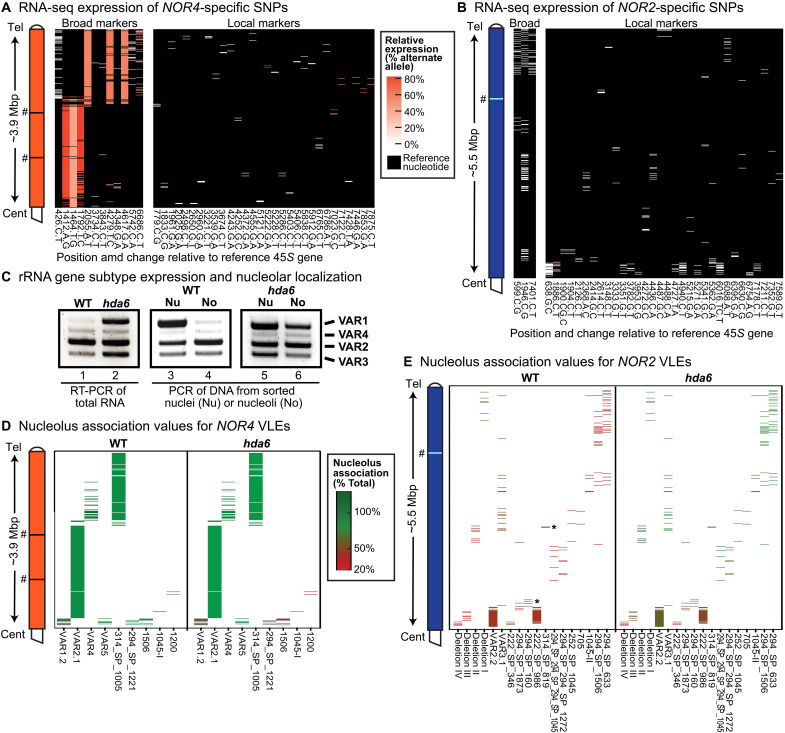
Ribosomal RNA (rRNA) gene activity and nucleolar association maps for *NOR2* and *NOR4*. (**A** and **B**) Heatmaps of expression levels for *NOR4*- or *NOR2*-specific single-nucleotide polymorphisms (SNPs), each represented by a horizontal line on the *y* axis. SNP names on the *x* axis include the position (number) in the ribosomal gene reference sequence followed by the reference and alternate nucleotide. White to red colors indicate level of expression based on normalized RNA sequencing (RNA-seq) read counts. Black indicates reference nucleotide positions with no alternative SNPs. (**C**) Reverse transcription polymerase chain reaction (RT-PCR) of the 3′ external transcribed spacer (3′ETS) VAR region, comparing wild-type (WT) and *hda6* mutants (left) and comparing nuclear- versus nucleolus-associated genes (middle and right). (**D** and **E**) Heatmaps depicting degree of nucleolus association for *NOR4*- or *NOR2*-specific variable-length elements (VLEs). Asterisks denote rare *NOR2* VLEs that are nucleolus associated. Experiments were performed using leaf tissue of mature plants. # symbols indicate positions in the assembly where contigs were joined manually, as described for Fig. 2D.

To determine where active rRNA genes are located within the NORs, we first identified informative nucleotide polymorphisms using Illumina whole-genome sequencing, conducted at a depth of coverage of ~163× (fig. S6A and data file S10) and determined the positions of these polymorphisms in our NOR assemblies (fig. S6B). In agreement with a prior study (*21*), polymorphic nucleotides are most abundant outside of the 18*S*, 5.8*S*, or 25*S* catalytic rRNA sequences incorporated into ribosomes (fig. S6A). We then performed Illumina sequencing of reverse-transcribed leaf RNA and identified polymorphic nucleotides within this read set. Of the 42 nucleotide polymorphisms specific to the transcribed regions of *NOR4* genes, 14 were detected by RNA sequencing (RNA-seq). Some are common to genes broadly distributed throughout the NOR, any of which could be responsible for the transcripts detected, such that these polymorphisms do not allow pinpointing of the genes that generated the transcripts. However, other polymorphisms localize to specific regions within the NOR. Collectively, the regions where there is overlap between the broad and region-specific polymorphisms that are detected in RNA indicate that genes in the central region of *NOR4* are most highly expressed. At the telomeric and centromere-proximal ends of *NOR4*, the polymorphic markers specific to these regions are not detected in the RNA, suggesting that transcriptional activity wanes near the NOR ends (Fig. 4A).

Thirty-five polymorphisms were specific to the transcribed regions of *NOR2* genes, representing gene subtypes distributed throughout the NOR (Fig. 4B). Only one was detected among expressed RNAs, C1886T, which is present within a cluster of genes near the NOR’s centromere-proximal end, indicating that *NOR2* is mostly, but not entirely, inactive.

### rRNA genes distributed throughout NOR4 copurify with nucleoli

The abundant nucleolar protein, Fibrillarin, when expressed as a fusion protein in-frame with yellow fluorescent protein (YFP), enables the fluorescence-activated flow-sorting of intact nuclei, liberated by cell disruption, as well as the sorting of nucleoli, which persist upon disruption of nuclei by sonication (*19*). Using a polymerase chain reaction (PCR) primer pair that flanks the 3′ETS variable region, amplification products of genes bearing the VAR1, VAR2, VAR3, or VAR4 VLEs (*20*) are all detected in the DNA of nuclei (Fig. 4C, lanes 3 and 5). However, VAR1 class genes are depleted in isolated nucleoli (Fig. 4C, lane 4), reflecting their selective silencing (Fig. 4C, lane 1). However, if silencing is prevented, in a mutant lacking histone deacetylase 6, VAR1 genes are expressed at high levels (Fig. 4C, lane 2) (*18*, *19*), and they now copurify with nucleoli (Fig. 4C, lane 6). These experiments show that nucleolar localization can be used as a proxy for rRNA gene transcriptional activity. This allows active genes to be identified on the basis of VLEs in their DNA sequences, thereby allowing detection of active rRNA gene subtypes whose transcripts may not have unique polymorphisms that enable them to be distinguished by RNA-seq.

Using ONT sequencing of DNA, we identified the rRNA gene subtypes that copurify with flow-sorted nucleoli or whole nuclei (as controls). The ONT reads averaged ~3 kbp in length (table S2), sufficient for VLE identification. The abundance of 3′ETS VLE reads from nuclear DNA matched their abundance in the NOR assemblies (fig. S7), indicating that all subtypes are detected, without apparent bias. Next, we calculated the level of nucleolar enrichment for NOR-specific VLEs. *NOR4* rRNA genes are enriched in nucleoli, as demonstrated for nine *NOR4*-specific VLEs (Fig. 4D), two of which (VAR2.1 and 314_SP_1005) represent homogeneous regions C and D (see Fig. 2, D and E). Nucleolar association of the *NOR4* VLEs was mostly unchanged in an *hda6* mutant versus wild type, except for decreased expression of genes near the centromere-proximal end of the NOR. By contrast, dramatic differences were apparent for *NOR2-*specific VLEs in wild type versus *hda6* (Fig. 4E). In wild-type plants, 16 of the 18 VLEs examined, distributed throughout *NOR2*, are depleted in nucleoli relative to nuclei but become nucleolus-enriched in *hda6* plants, indicative of HDA6-dependent silencing. Notably, genes near the centromere-proximal end of *NOR2* failed to become nucleolus-enriched in *hda6* plants. Many genes in this region are missing promoters (see Fig. 3F), likely precluding their participation in nucleolus formation. Two of the 18 *NOR2*-specific VLEs were nucleolus-associated (see asterisks in Fig. 4E) even in wild type, with one present in a small gene cluster near a cluster also identified by RNA-seq (Fig. 4B), consistent with pockets of gene expression within *NOR2*.

### NOR2 and NOR4 have distinct large-scale cytosine methylation landscapes

**Fig. 5.**
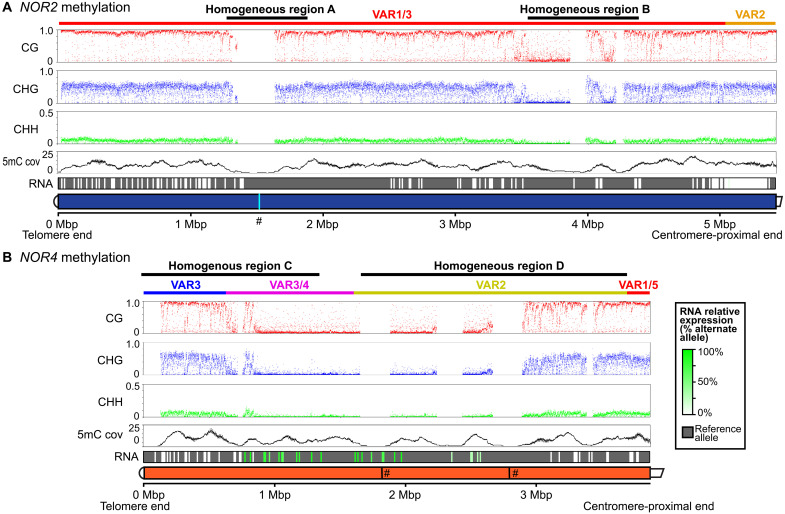
Methylation landscapes of *NOR2* and *NOR4*. (**A** and **B**) 5-methylcytosine (5mC) frequencies in the CG, CHG, and CHH contexts at *NOR2* and *NOR4*. Each colored point represents the frequency calculated within a 500-bp sliding window, with 250-bp steps. Homogeneous regions where there are no data points reflect a paucity of uniquely mapped reads needed to surpass the threshold for 5mC calling. The 5mC cov (coverage) track shows the number of reads that were retained by the methylation calling pipeline. The RNA track shows the relative expression levels of single-nucleotide polymorphisms (SNPs), colored from white (not expressed) to shades of green (expressed). The gray background corresponds to nucleotides of the reference sequence. The DNA sequenced was from inflorescence apex tissue. # symbols indicate positions in the assembly where contigs were joined manually, as described for Fig. 2D.

We examined cytosine methylation in the CG, CHG, and CHH (where H is a nucleotide other than G) sequence contexts throughout *NOR2* and *NOR4* using ONT methylation calling tools (*32*), calculating the percent methylation within 500-bp sliding windows (Fig. 5, A and B). Most of *NOR2* has extremely high CG methylation, moderate CHG methylation, and relatively low CHH methylation (Fig. 5A). An exception is *NOR2* homogeneous region B, which is hypomethylated in all three contexts (Fig. 5A and fig. S8). At *NOR4*, a central region spanning ~2 Mbp has low methylation levels, in all contexts, but is flanked, on both sides, by ~1-Mbp intervals of high methylation (Fig. 5D). The most highly methylated regions of *NOR2* and *NOR4* correlate with regions of low or undetectable gene expression (Fig. 5, A and B, RNA track). Conversely, the hypomethylated central region of *NOR4* overlaps the positions of actively transcribed gene subtypes (Fig. 5B, RNA track).

Zooming in within regions of high CG methylation, we observe that methylation is high across entire gene units except near the gene promoter, which is hypomethylated in most, but not all, genes (fig. S8). Conversely, in low methylation regions, the hypomethylation is uniform except in the 3′ETS region, where a peak of methylation occurs in most gene repeats (fig. S8). The meaning of these distinctive methylation signatures is unclear, but their occurrence at the beginnings and ends of rRNA gene transcription units is intriguing, suggesting potential regulatory significance.

## DISCUSSION

Using an unconventional sequence assembly strategy, our determination of the *NOR2* and *NOR4* sequences, from their telomere-capped ends to their centromere-proximal ends, fill the last major gaps that remain in the Arabidopsis genome following the determination of the centromeric regions (*26*). The two NOR sequences add 9.3 Mbp of genome sequence information, equivalent to ~6.5% of the genome.

A surprising finding is that none of the 74 gene subtypes defined by VLE content is common to both NORs. Instead, the NORs have unique sets of rRNA gene subtypes, allowing subtype-specific expression to be mapped to specific positions within *NOR2* or *NOR4*. Collectively, the data show that genes distributed throughout the 5.5 Mbp of *NOR2* are inactive, with only rare exceptions, whereas genes throughout a large central region of *NOR4* are expressed. Inactive *NOR2*-specific gene subtypes are epigenetically silenced, in an *HDA6*-dependent manner (Fig. 4). Collectively, these analyses of rRNA gene activity and epigenetic regulation provide a comprehensive view of nucleolar dominance as it occurs on the scale of whole NORs, with neither NOR being fully active nor completely silenced.

Prior studies showed that silencing of rRNA genes with VAR1 class 3′ETS sequences, which primarily map to NOR2 (*20*), requires the DNA methyltransferases MET1 and CMT2, in addition to HDA6 and histone H3 lysine 9 methyltransferases (*16*, *19*, *33*, *34*). Another study showed that changes in histone posttranslational modifications and DNA methylation occur in a concerted manner at both silenced and active rRNA genes (*35*). These findings provide context for the ~2-Mbp cytosine-hypomethylated region at the center of *NOR4*, which overlaps the positions of rRNA gene subtypes that are transcribed and associated with the nucleolus. Our interpretation is that this hypomethylated region is the likely source of most rRNAs synthesized in the leaf and inflorescence tissues used in our study. Our observation that *NOR4* gene subtype expression is mostly unchanged in *hda6* plant*s* (relative to wild type), unlike *NOR2* gene subtypes, suggests that the lower levels of gene expression observed near the telomere- and centromere-proximal ends of *NOR4* reflect regulatory mechanisms distinct from those involved in *NOR2* gene silencing.

Another intriguing finding of our study is that regions of high gene activity and/or low cytosine methylation within *NOR2* and *NOR4* correlate with regions of high gene subtype homogeneity. Concerted evolution of rRNA genes (*36*, *37*) is thought to occur via gene conversion or recombination following slipped mispairing of NOR sequences (*38*). Our results suggest that transcription may facilitate local rRNA gene homogenization via one or both mechanisms. Consistent with this hypothesis, studies in *Saccharomyces cerevisiae* have shown that mitotic recombination and gene conversion events are stimulated by rRNA gene promoter sequences (*39*–*41*). Studies in yeast also demonstrated that unequal exchanges occur between the NORs of sister chromatids at meiosis (*42*), suggesting a mechanism whereby repeated exchanges of whole rRNA gene units or clusters of identical units could become homogenized at specific locations within a NOR. However, our finding that no gene subtypes are common to both *NOR2* and *NOR4* argues against exchanges of complete rRNA genes between the NORs as an explanation for how concerted evolution occurs within the species. Instead, sequences that are shared by the genes of both NORs are limited to the scale of VLEs (see fig. S5), suggesting that coevolution of the genes within the two NORs occurs through events involving sequence intervals shorter than complete gene units.

The correlation between hotspots of transcription and hotspots of gene subtype homogenization is consistent with transcription acting as a driver of homogenization, perhaps explaining how two gene subtypes of *NOR4* have come to occupy 82% of the NOR. Conversely, the substantial heterogeneity *NOR2* (47 subtypes versus 27 at *NOR4*), including the persistence of genes with debilitating structural anomalies, may reflect its reduced transcriptional activity. Future studies of *A. thaliana* strains in which the dominance relationships between *NOR2* and *NOR4* are reversed relative to the situation in Col-0, or in which the two NORs are codominant (*21*), should allow tests of this hypothesis.

## MATERIALS AND METHODS

### Experimental design

The objectives of the study were to obtain gapless sequences for *NOR2* and *NOR4* in the *A. thaliana* strain Col-0 to map their functional organizations and determine the full scope of nucleolar dominance across the entirety of the NORs. Our approach was to use the NOR sequences to define the gene subtype compositions of the NORs, determine the locations of active gene subtypes and of inactive subtypes that are epigenetically silenced in an HDA6-dependent manner, and also determine how cytosine methylation patterns relate to the gene activity maps.

### Plant material

*A. thaliana* Col-0 (Arabidopsis Biological Resource Center stock CS1092), Col-0 bearing the Fib2:YFP transgene (*43*), and Col-0 Fib2:YFP, *hda6-6* mutant plants (*19*) were the sources of DNA or RNA used in this study. For 3-week-old seedling tissue, plants were grown on 0.5× Murashige and Skoog agarose plates. For inflorescence apex and leaf tissue, plants were grown in soil.

### PacBio SMRT sequencing

Six grams of frozen inflorescence apex tissue was used for cetyltrimethylammonium bromide (CTAB)/phenol/chloroform DNA extraction (for details, see the DNA extraction section of https://benchling.com/s/prt-lQHskqJ9QFYOJ1MWhMs4?m=slm-3raRnwghjJbspfwq3k9Z). The resulting gDNA was digested with I-Ppo I to cut 45*S* rRNA gene arrays into individual units averaging ~10 kbp. The digested DNA was subjected to electrophoresis using a 0.7% agarose gel, and the ~10-kb region was excised and purified using the Qiagen’s Qiaex II Gel Extraction Kit. Libraries were prepared from this DNA using Pacific Bioscience’s DNA Sequencing Kit 4.0, and sequencing was conducted using a PacBio RS II system.

### Analysis of SMRT sequencing data to determine IGS VLE types

Raw PacBio subreads were placed into the ccs command of the SMRT tools 9 package <ccs --minReadScore=0.6 --minLength=5000 --maxLength=13000 --minPasses=1 --minPredictedAccuracy=0.85 $input $output> to generate consensus read sequences. These reads were aligned to the 45*S* IGS reference sequence used in a prior study (*30*). Aligned sequences that showed significant structural differences to the reference were manually annotated to generate reference sequences representing a broad set of 45*S* IGS sequence types. Additional IGS sequences were discovered during subsequent ONT sequencing data assembly and added to the set of 45*S* IGS sequence types.

### Ultralong ONT sequencing

gDNA from Col-0 plants was extracted using Circulomics Nanobind products and protocols (https://pacb.com/support/documentation/?fwp_documentation_search=plant&fwp_workflow_step=sample-preparation&fwp_sort=preserve). For each prep, 350 to 1000 mg of frozen inflorescence apex tissue (unopened flower buds) were processed using the Circulomics’ “Nuclei Isolation – TissueRuptor Plant Tissue Protocol” (document ID: NUC-TRP-001). High molecular weight DNA was then extracted from the resulting nuclei preparations using Circulomics’ “Nanobind HMW DNA Extraction – Plant Nuclei Protocol” (document ID: EXT-PLH-002). Libraries were prepared using 40 μg of resulting DNA (measured using a Qubit fluorometer) and an ultralong DNA sequencing kit (SQL-ULK001). Sequencing was performed for 72 hours using either a MinION or GridION sequencer with R9.4.1 flowcells.

### Whole-genome assembly

Base-calling of raw ONT sequencing data was performed using Guppy 6.1.2 using the psac (plant super accurate) model. Only reads below 100 kbp were retained. Filtlong (https://github.com/rrwick/Filtlong) was then used to sample reads to ~30× coverage (filtlong --min_length 1000 --mean_q_weight 5 --target_bases 4200000000 $LONG_READ > ${LONG_READ%.*}_filtlong_30Xquality.fq). Filtered reads were then assembled by canu 2.1 (*44*) (/canu-2.1/bin/canu -p $NAME -d $outpath genomeSize=140 m -nanopore $infile useGrid=false maxThreads=24 maxMemory=210 overlapper=mhap utgReAlign=true). The resulting assembly was compared against the TAIR10 nuclear genome using RagTag (*45*) and visualized using Assemblytics (*46*).

### rRNA gene assembly using dot plots

For similarity matrix (dot plot)–based assembly, base-calling of raw ONT sequencing data was performed with Guppy 3.6.1. 45*S* rRNA gene–containing reads were selected by mapping fastq reads to the consensus rRNA gene reference using minimap2 (*47*). Dot plots were generated with the dottup tool (word size = 11) of Geneious 9.0.5. and joined based on overlapping, visually distinct patterns.

### NOR assembly computational pipeline

Base-calling of raw ONT sequencing data was performed using Guppy 6.1.2 using the psac (plant super accurate) model. Filtlong was used to first select reads longer than 35 kbp. Reads that mapped to the Col-0 45*S* rRNA gene consensus upon minimap2 alignment were then selected. To retain the longest reads, 45*S* rRNA gene reads over 275 kbp were saved and set aside, while the remaining reads were put through additional selection steps, first splitting reads at locations with no NOR kmers for more than 12 kbp (filtlong -a references.fa --split 12000) and then filtering for high-quality, long reads of at least 85 kbp (filtlong --min_length 85000 --min_mean_q 90). This selected, quality-controlled 45*S* rRNA gene read set was then concatenated with the unfiltered >275-kbp 45*S* rRNA gene read set, resulting in the final NOR assembly read set.

Computational assembly involved the creation of two Snakemake-based pipelines (https://snakemake.readthedocs.io/en/stable/): perread_var_call and tagged_longread_assembler, which we have made publicly available at Zenodo (https://zenodo.org/record/8335855). First, the NOR assembly read set was converted into “VLE sequence” using the perread_var_call. Briefly, the pipeline split reads into segments starting at 25*S* rRNA sequences. These segments were then mapped, via minimap2, to each known variant sequence for the different VLE regions. The element variant with the highest minimap2 alignment score was then chosen as the “variant call” for that VLE. This was repeated for each VLE. The resulting variant calls throughout a sequencing read were then collected in order, creating a VLE sequence for that read. Second, VLE sequences were used to generate maps of the NORs. This was done using scripts from the tagged_longread_assembler repository. Starting from the NOR telomeric and centromere-proximal ends, VLE strings with unique patterns (VLE landmarks) were gathered and visualized using the “single-pattern_to_SValignment.py” script. Using these visualizations, additional VLE landmarks were discovered, confirmed by analysis of their coverage, and “maps” of the distance between VLE landmarks were created. These maps were confirmed using visualizations of alignments of the VLE sequences generated by “locus-patterns_to_SValignment.py” and color-coded using “SValign_to_colored-xlsx.py.” These visualizations are provided in data files S3 to S7. This process allowed the linking of VLE landmarks into contigs across the entirety of both NORs. Third, DNA sequence assembly was performed using the main “tagged_longread_assembler” pipeline. The map files for each contig, combined with the NOR assemblies read set and VLE sequence files, were used as input to generate polished contigs. Briefly, the pipeline searched the VLE sequences for the VLE landmarks of the map. It then inserted a unique DNA sequence tag at each 25*S* sequence in the original fastq reads, defining where that portion of the read occurs in the NOR. This allowed all read segments from a given read to be placed into a multiple sequence alignment with other reads corresponding to the same position within a NOR. The resulting consensus sequences for each position were then joined into a contig. The tagged reads were then used for several rounds of Racon polishing, with the unique sequence tags forcing proper alignment. The pipeline then removed the unique DNA tags, resulting in polished contigs (see the “tagged_longread_assembler” github for further details). Last, the polished contigs were merged by hand. Low coverage regions at ends of contigs were trimmed.

At NOR ends, where the NORs join either the telomeres or centromere-proximal non-rRNA gene sequences, junction sequences were merged manually. Sites where overlapping/adjacent NOR contigs could not be uniquely, computationally joined due to extensive gene subtype homogeneity (e.g., sites denoted by # in the figures) were also joined manually, guided by subtype copy number estimates. Resulting final sequences for *NOR2* and *NOR4* were annotated using Geneious Prime and submitted to GenBank.

### Analysis of assembled NOR sequences

*NOR2* and *NOR4* fasta files were placed into the “perread_var_call” pipeline, yielding VLE variant calls for the assembly. The script “SV-kmer_homogeneity.py” from “perread_var_call” was used to analyze the homogeneity of the NORs within a sliding window spanning nine rRNA genes. RStudio was used to generate color-coded tracks displaying the positions of VLE variant calls and homogeneity scores along the NORs. The script “SV-kmer_dot-plot.py” from “perread_var_call” was used to generate a similarity matrix using VLE sequence rather than DNA sequence. RStudio was used to display the output. Similarity matrix analyses comparing NOR VLE patterns to a 45*S* rRNA gene reference VLE pattern were used to visualize the direction and organization of the repeats.

### sgRNAs for targeted Cas9 cutting in the 3′ETS region of different rRNA gene subtypes

We used a custom script to identify potential target sequences present in the 3′ETS variable region, following guidelines for single-guide RNA (sgRNA) design (http://clontech.com/sgRNA-design-tools). A set of forward PCR primers were then made that included T7 promoter sequences adjacent to the sgRNA target sequences and the Guide-it Scaffold Template–specific sequence for sgRNA synthesis using the Guide-it sgRNA In Vitro Transcription Kit (catalog no. 632635). RNA products were purified using a Guide-it IVT RNA Clean-Up Kit (catalog no. 632638). The efficacy of the different sgRNAs was then tested using a Guide-it sgRNA Screening Kit (catalog no. 632639). Briefly, gDNA (50 ng) purified from Col-0 plants was incubated with sgRNA (25 ng) in the presence of 250 ng of Cas9 nuclease. The Cas9 cleavage efficiency within the 3′ETS region was then assessed by PCR amplification using a pair of primers flanking the 3′ETS region (forward: GACAGACTTGTCCAAAACGCCCACC; reverse: CTGGTCGAGCTAATCCTGGACGATT). Reactions were then subjected to agarose gel electrophoresis and SYBR Safe staining. The sgRNAs ultimately used in the study (see fig. S4) and their 3′ETS VLE specificities were VAR1 + VAR2–targeting sgRNA, ATGAAAACTGGTGATTGTTGCGG; VAR1 + VAR3– targeting sgRNA, AGAAACGGAAGAGAAAGCGTGGG; and VAR1, VAR3, VAR4–targeting sgRNA, GAACTAGCAAGTAATCGTCCAGG.

### Analysis of genomic sgRNA-Cas9 digestion by CHEF gel electrophoresis and Southern blotting

Ultrahigh molecular weight DNA was prepared as described for ONT sequencing and embedded in agarose plugs. Agarose plugs were then placed in 50-ml conical tubes and incubated in 10 ml of T10E10 buffer [10 mM tris-HCl and 10 mM EDTA (pH 8.0)] supplemented with 2 mM phenylmethylsulfonyl fluoride (PMSF) for 1 hour at 4°C. Plugs were then washed four times, 30 min each, at room temperature in 10 ml of T10E10 buffer without PMSF. Next, individual agarose plugs were washed twice, 1 hour, at room temperature, with 1 ml of 1× Cas9 reaction buffer [New England Biolabs (NEB), catalog no. B0386]. After a second wash, the plug was incubated for 1 hour, at 37°C, with 100 μl of 1× Cas9 reaction buffer (NEB) containing 200 μM sgRNAs and 1 μM Cas9 enzyme. The buffer was then removed and replaced with 500 μl of 20 mM tris-HCl, 50 mM EDTA (pH 8.0) and incubated for 10 min at room temperature. The agarose plug was then subjected to five washes, each 15 min at room temperature, with 10 ml of T10E10 buffer. Agarose plugs were inserted into the wells of a precast 1% Certified Megabase Agarose (Bio-Rad) gel and subjected to CHEF electrophoresis using a Bio-Rad system. Running parameters for stage I were initial and final switch time of 60 s, 200 V, and run time of 13 hours, and running parameters for stage II were initial and final switch time of 90 s, 200 V, and run time of 7 hours. The gel was then subjected to Southern blotting as described by Mohannath and Pikaard (*48*). The digoxigenin (Dig)–labeled 25*S* rDNA probe was synthesized using a PCR Dig Probe Synthesis kit (Roche, 11636090910) and primers CCGGAGGTAGGGTCCAGCGG (forward) and CCGCCGTTTACCCGCGCTTG (reverse). Hybridization was performed at 42°C, for 72 hours, followed by membrane washing and blocking using Dig Wash and Block Buffer Set (Roche, 11585762001). rRNA gene fragments were detected using anti-Dig AP antibodies (1:15,000 dilution) (Roche, 11093274910) and CDP-Star Detection (Roche, 11759051001).

### Whole-genome Illumina sequencing

gDNA was isolated from Arabidopsis seedlings using the Qiagen DNeasy Plant Mini Kit (catalog no. 69106). The gDNA library was prepared using the Illumina Nextera Flex Kit (catalog no. 20025523) and then sequenced on an Illumina NextSeq instrument to obtain 75-bp paired-end reads. The resulting sequence data were subjected to quality control and trimming using Trimmomatic (https://github.com/usadellab/Trimmomatic). The data were then mapped to a 45*S* rDNA consensus sequence using Bowtie2, with allowance for a single mismatch in the seed region. The mapped reads were variant-called using LoFreq (*49*) (https://csb5.github.io/lofreq/), which generated a Variant Call Format (VCF) file with all the variant calls across a single rDNA unit. The --no-default-filter flag was used to run LoFreq with high sensitivity and then filtered for sites predicted to occur with a frequency higher than 0.001. The resulting VCF file was input into the custom snakemake pipeline “perread_SNP_call” along with the NOR assembly sequences. Briefly, the first portion of this pipeline maps each unit of the input sequences (NOR assembly) to the reference (45*S* reference) and then looks at the mapped bases at each position of interest (as defined by the LoFreq vcf), outputting a line of variant calls for each unit. RStudio was used to generate a heatmap display of reference versus variant nucleotides positioned along the NORs.

### RNA sequencing

Mature leaf of 3-week-old plants or inflorescence tissue was collected, and total RNA was extracted using the Zymo Research Quick-RNA Plant Miniprep Kit (catalog no. 11-362). The resulting RNA was treated with Turbo DNase (catalog no. AM2238). The library was prepared using an Illumina TruSeq Stranded Total RNA HT kit with no enrichment or depletion steps. The library was sequenced on an Illumina NextSeq instrument to obtain 75-bp paired-end reads. Resulting sequence data were subjected to quality control and trimmed using Trimmomatic. The data were mapped to 45*S* rDNA consensus sequence using Bowtie2, allowing a single mismatch in the seed region. The mapped reads were then filtered for those that mapped in the forward direction, and these were then variant-called using LoFreq with the --no-default-filter flag to maximize sensitivity. The resulting VCF files were input into the second portion of the “perread_SNP_call” pipeline. This takes the alternate allele frequency from the RNA-seq vcf, normalized the values based on the number of occurrences of that allele in the assembly, and then output the normalized values at the location of the variant allele (polymorphism relative to the reference sequence) on the NORs. The resulting normalized expression values were displayed in a heatmap using RStudio.

### Flow sorting of nuclei and nucleoli, DNA isolation, and sequencing

To purify nuclei, ~2 g of *A. thaliana* leaf tissue from ~3-week-old seedlings (wild-type or *hda6* mutant Col-0 expressing Fib2:YFP) was fixed for 20 min in 4% formaldehyde in tris buffer [10 mM tris-HCl (pH 7.5), 10 mM EDTA, and 100 mM NaCl], washed twice for 10 min with ice-cold tris buffer, and minced with a razor blade in 1 ml of 45 mM MgCl_2_, 20 mM MOPS (pH 7.0), 30 mM sodium citrate, and 0.1% Triton X-100. The homogenate was filtered through a 30-μm mesh (Sysmex CellTrics) and subjected to fluorescence-activated nuclear sorting, triggered by the Fib2:YFP signal, using a BD FACSAria II instrument as described previously (*19*). To purify nucleoli, the homogenate was subjected to sonication with the Diagenode Bioruptor (three 5-min pulses; medium power), which disrupts nuclei. The sonicated material was then subjected to fluorescence-activated nucleolar sorting (FANoS) (*19*). Approximately 1 million sorted nuclei and ~1.5 million sorted nucleoli were collected. The nuclei and nucleoli samples were then treated with proteinase K [100 μl of stock solution (20 mg/ml)/1 ml of sample] and ribonuclease A [20 μl of stock solution (10 mg/ml)/1 ml of sample]. After gentle mixing by tube inversion, the samples were incubated at 50°C for 30 min, followed by incubation at 95°C for 20 min. Two hundred fifty microliters of 4× DNA extraction buffer [0.55 M tris-HCl (pH 8.0), 1 M NaCl, and 100 mM EDTA] was then added per 1 ml of final sample volume and mixed gently by tube inversion. DNA was precipitated by the addition of 1 volume of ice-cold isopropanol and incubation on ice for 1 hour in the presence of 1 μl (15 μg/μl) of Glycoblue. DNA was recovered by centrifugation of 16,000*g* for 15 min at 4°C. The DNA pellet was washed twice with 1 ml of ice-cold 70% ethanol and resuspended in 1× TE buffer. DNA libraries were prepared using the ONT Rapid Sequencing Kit (SQK-RAD004) and sequenced using a GridION sequencer with R9.4.1 flowcells.

### Analysis of 5mC positions in ONT sequencing data

Detection of 5-methylcytosine (5mC) modification was performed using deepsignal-plant (*32*), as described in the “Usage” section of the associated GitHub (https://github.com/PengNi/deepsignal-plant), using raw sequencing data (fast5s) corresponding to the fastq reads used for the NOR assemblies. This was followed by quantification of methylcytosine frequency according to sequence context (CG, CHG, or CHH) using the included Python script. The resulting 5mC frequencies for each cytosine in the NOR assemblies were binned using sliding windows of 500 bp, with a 250-bp step. Resulting values were then imaged in *xy* plots using RStudio.
