# Supplementary material for: Sequence and epigenetic landscapes of active and silent nucleolus organizer regions in Arabidopsis
Source: Sci Adv. 2023 Nov 1;9(44):eadj4509. doi: 10.1126/sciadv.adj4509 (PMC10619934; doi:10.1126/sciadv.adj4509)
Supplement: Supplementary file 1 — Figs. S1 to S8 Tables S1 and S2 Legends for data S1 to S10 [file sciadv.adj4509_sm.pdf]

Science Advances
AAAS

Supplementary Materials for

Sequence and epigenetic landscapes of active and silent nucleolus organizer regions in *Arabidopsis*

Dalen Fultz *et al*.

Corresponding author: Craig S. Pikaard, cpikaard@iu.edu

Sci. Adv. **9**, eadj4509 (2023)
DOI: 10.1126/sciadv.adj4509

The PDF file includes:

Figs. S1 to S8
Tables S1 and S2
Legends for data S1 to S10

Other Supplementary Material for this manuscript includes the following:

Data S1 to S10

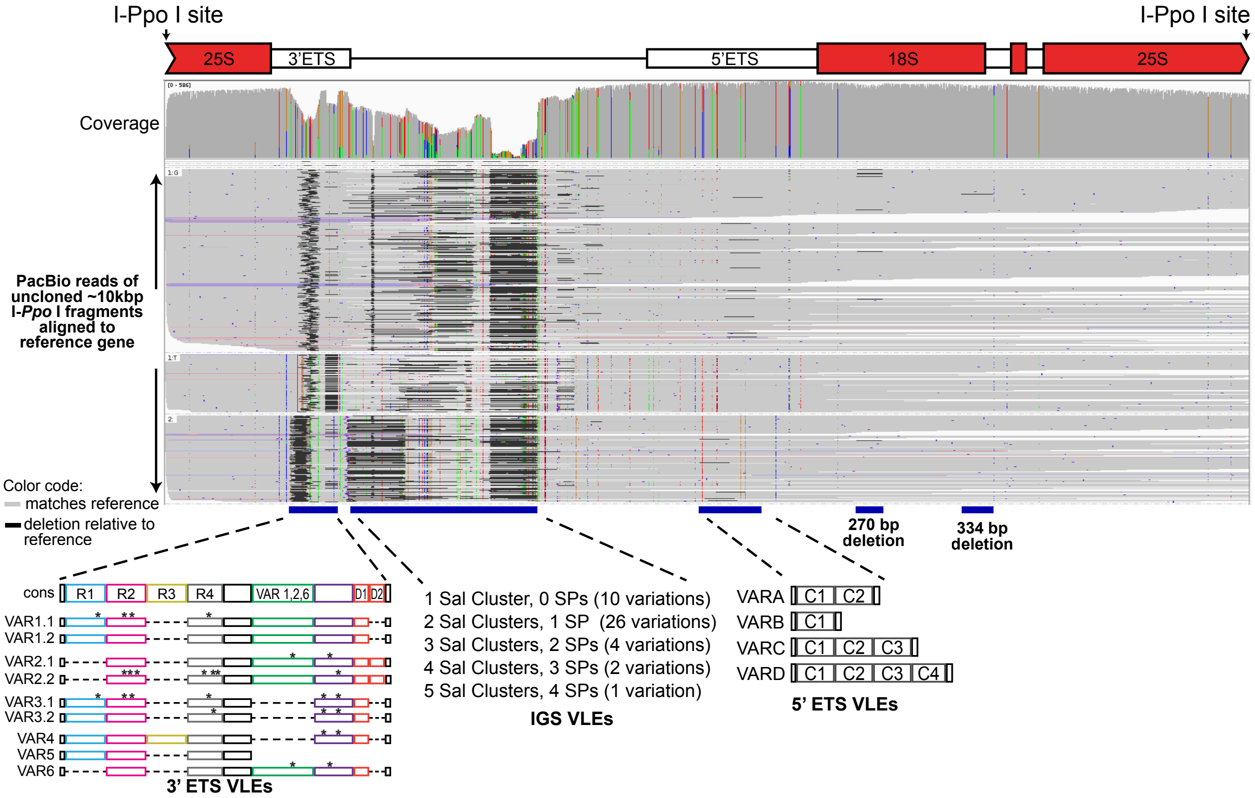

Fig. S1. Variation identified by PacBio sequencing of rRNA gene repeat units.

Genomic DNA was digested with the rDNA-specific endonuclease *I-Ppo* and resulting ~10 kb fragments were gel-purified and deep-sequenced. Individual reads were then aligned to an rRNA gene reference sequence (see file S1). Sequences identical to the reference are shown in gray. Differences relative to the reference are shown in black or other colors. Intervals corresponding to the VLEs used to define rRNA gene subtypes are shown at the bottom of the figure, as in Figure 1B.

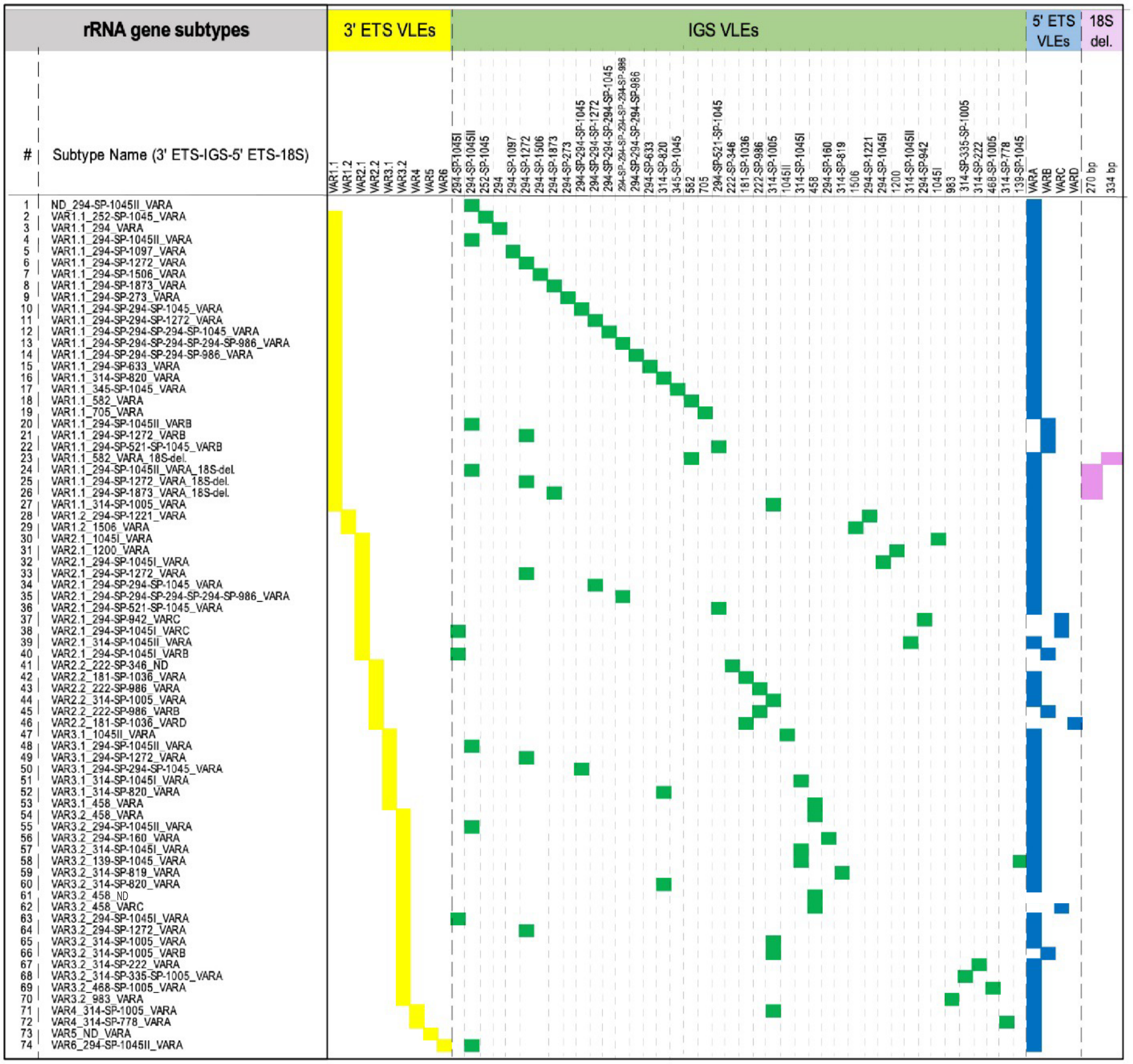

Fig. S2A. Gene subtypes defined by their VLE compositions.
The 59 VLEs summarized in Figure 1B occur in 74 different combinations among rRNA genes, thus defining 74 distinct rRNA gene subtypes. The most prevalent VLE is VARA, occurring in 64 gene subtypes and corresponding to the presence of two tandem C repeats within the 5' ETS. VLEs corresponding to different 3' ETS sequences provide the next largest groupings, with 27 gene subtypes having VAR1-class VLEs (with point mutations allowing 1.1 or 1.2 sub-classifications), twenty-four having VAR3.1 or 3.2 VLEs and nineteen having VAR2.1 or 2.2 VLEs. The least frequent subtypes carry VAR4, VAR5, and VAR6 VLEs. Subtype-specific VLEs occur primarily in the IGS, where there can be as few as one, or as many as five, clusters of Sal repeats (each repeat being 20-21 nt long) that vary in number. Sal repeat cluster lengths, in bp, are given in the gene subtype names. Likewise, variable numbers of spacer promoters (SPs) occur when there are two or more Sal repeat clusters. In total, there are 42 different Sal cluster-SP arrangements, 27 of which are unique to single rRNA gene subtypes. The remaining 15 IGS arrangements occur in two or more subtypes, an example being 294-SP-1272, having Sal repeat clusters of 294 bp and 1272 bp separated by a single spacer promoter. This IGS arrangement is present in six subtypes that collectively represent the 3 major 3'ETS classes, VAR1, VAR2 and VAR3.

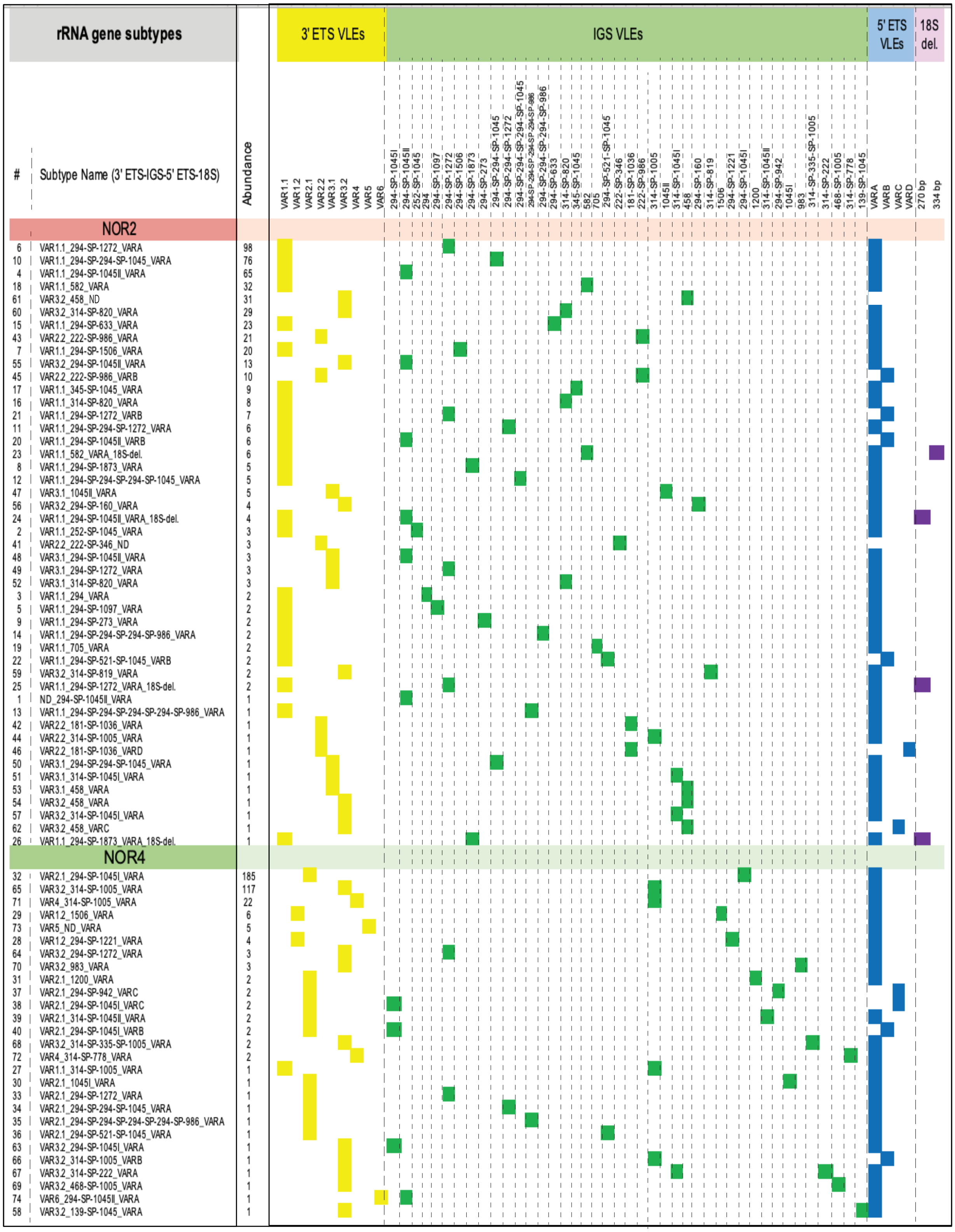

Fig. S2B. rRNA gene subtypes sorted according to *NOR2* or *NOR4* affiliation.

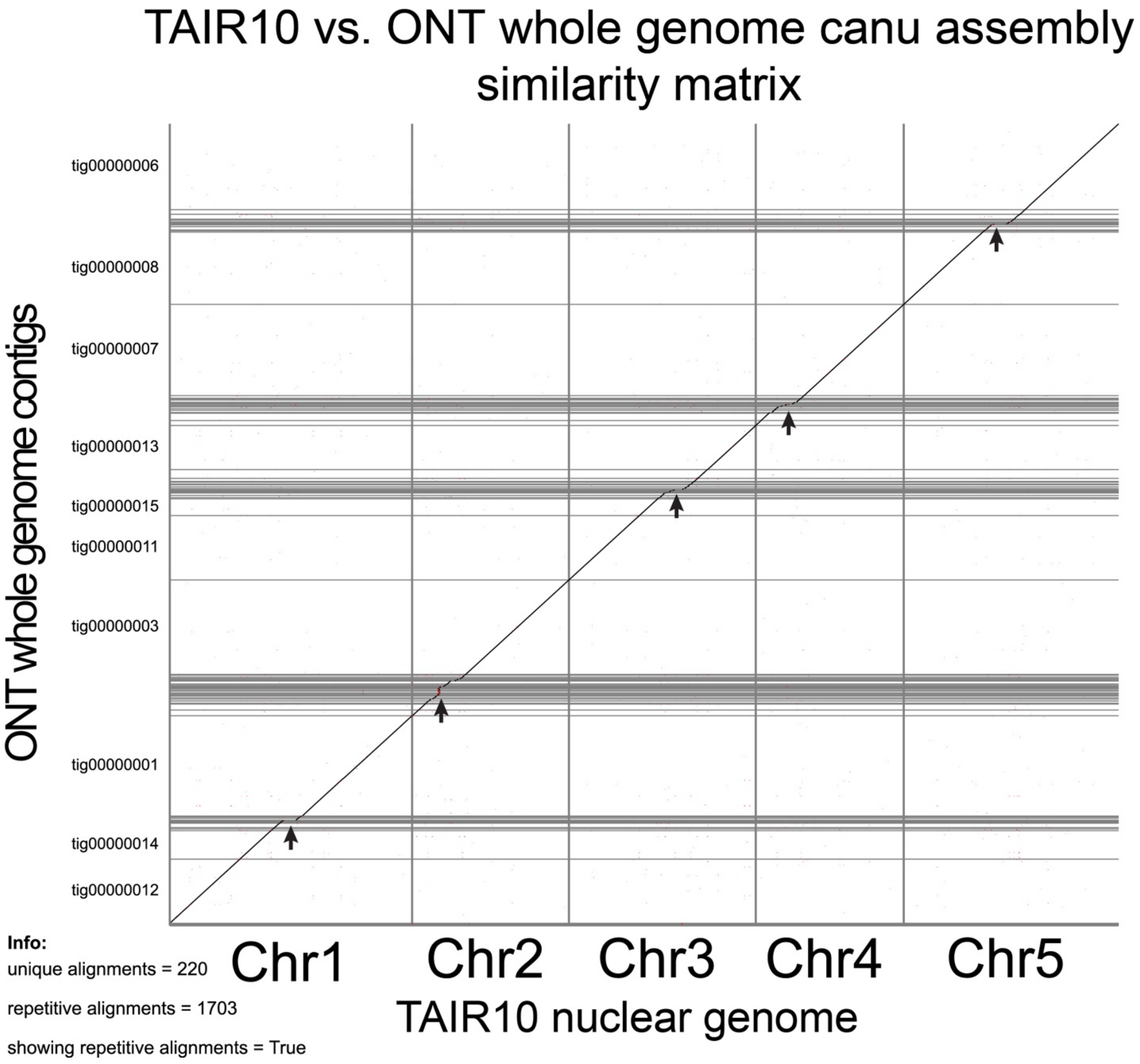

Fig. S3. Chromosome sequences assembled using ONT reads of the current study are contiguous with those of the TAIR10 assembly.

Contigs assembled using ultra-long ONT reads and Canu assembly tools (see methods) were scaffolded to the Arabidopsis nuclear chromosome assemblies from TAIR10 using RagTag. The similarity matrix was generated using the Assemblytics tool. The ONT sequence assembly aligns with the majority of the TAIR10 reference with no indicators of large rearrangements. Observed discontinuities, noted by arrows, occur in peri-centromeric regions.

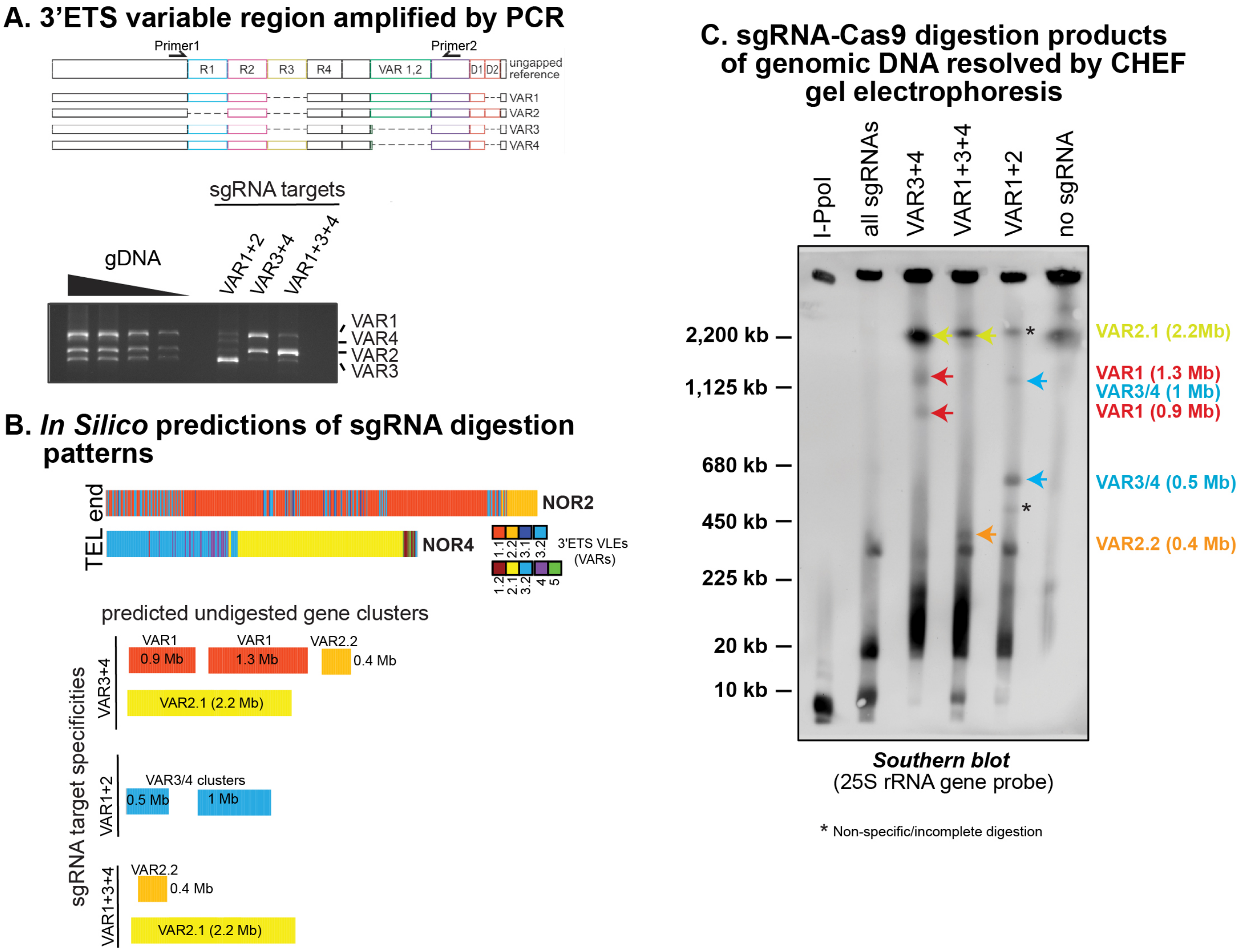

Fig. S4. Physical mapping test of the NOR assemblies using custom guide RNAs that direct Cas9 cleavage of rRNA gene 3' ETS regions.

(**A**) The diagram shows the 3'ETS variable region and the positions of PCR primers that flank the region. PCR amplification of genomic DNA using these primers yields products of different lengths, corresponding to the abundant genes bearing the VAR1, VAR2 and VAR3 VLEs and the less abundant genes bearing the VAR4 VLE. The stained agarose gel shows the amplification products obtained using either uncut genomic DNA (left lanes) or DNA that had been incubated with Cas9 and three different sgRNAs prior to PCR. Two of the sgRNAs guide the digestion of genes of two different VLE classes that share the same sgRNA target sequence (either VAR1 + VAR2 or VAR3 + VAR4), and the third sgRNA targets three VLE classes (VAR1 + VAR3 + VAR4). Note that the targeted VLE classes are depleted among the PCR amplification products, demonstrating the specificity and efficacy of the sgRNA-Cas9 complexes. (**B**) *In silico* prediction of large sgRNA-Cas9 digestion fragments of *NOR2* and *NOR4* based on the sgRNA specificities demonstrated in panel A. The sizes of the expected fragments are shown, with color-coding showing the regions of the NORs giving rise to the fragments. (**C**) sgRNA-Cas9 digestion products visualized by CHEF gel electrophoresis and Southern blotting with a 25S rRNA probe. For this experiment, ultra-high molecular weight gDNA was embedded in agarose plugs and subjected to Cas9 digestion programmed by individual sgRNAs, as in panel A, or a mix of all three sgRNAs. I-*Ppo*I and no-digestion controls are included in the first and last lanes. The DNA fragments were resolved by CHEF electrophoresis and visualized by Southern blotting and hybridization to the 25S rDNA probe. Predicted large fragments (see panel B) were observed.

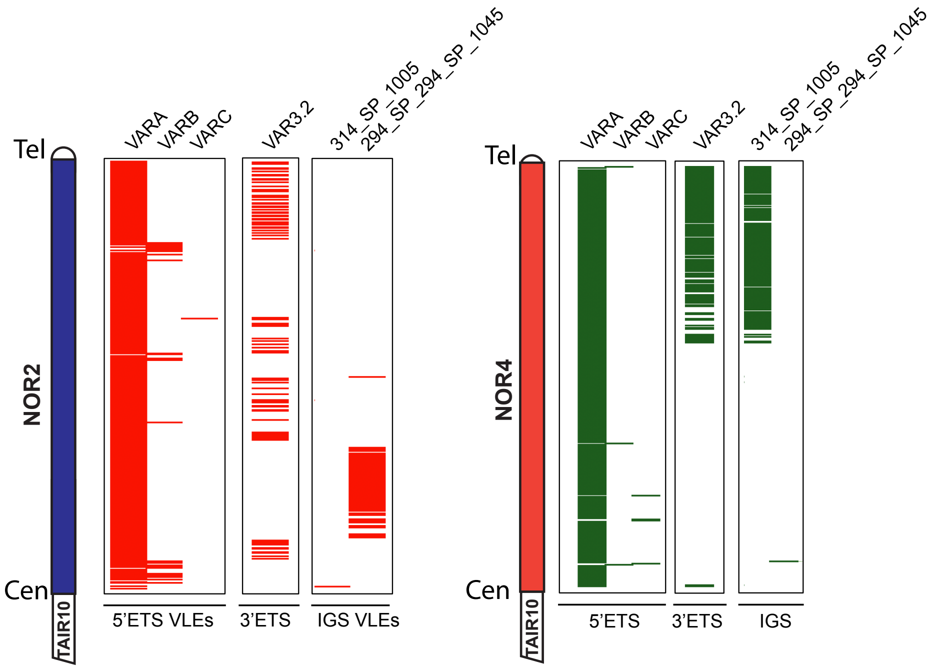

Fig. S5. VLEs that are common to both NORs.

The positions of 6 VLEs present within genes of both *NOR2* and *NOR4* are indicated by colored horizontal lines. Tel and Cen indicate the telomere and centromere-proximal ends of the NORs. TAIR10 indicates where the current sequences of chromosomes 2 and 4 begin in the TAIR10 genome assembly.

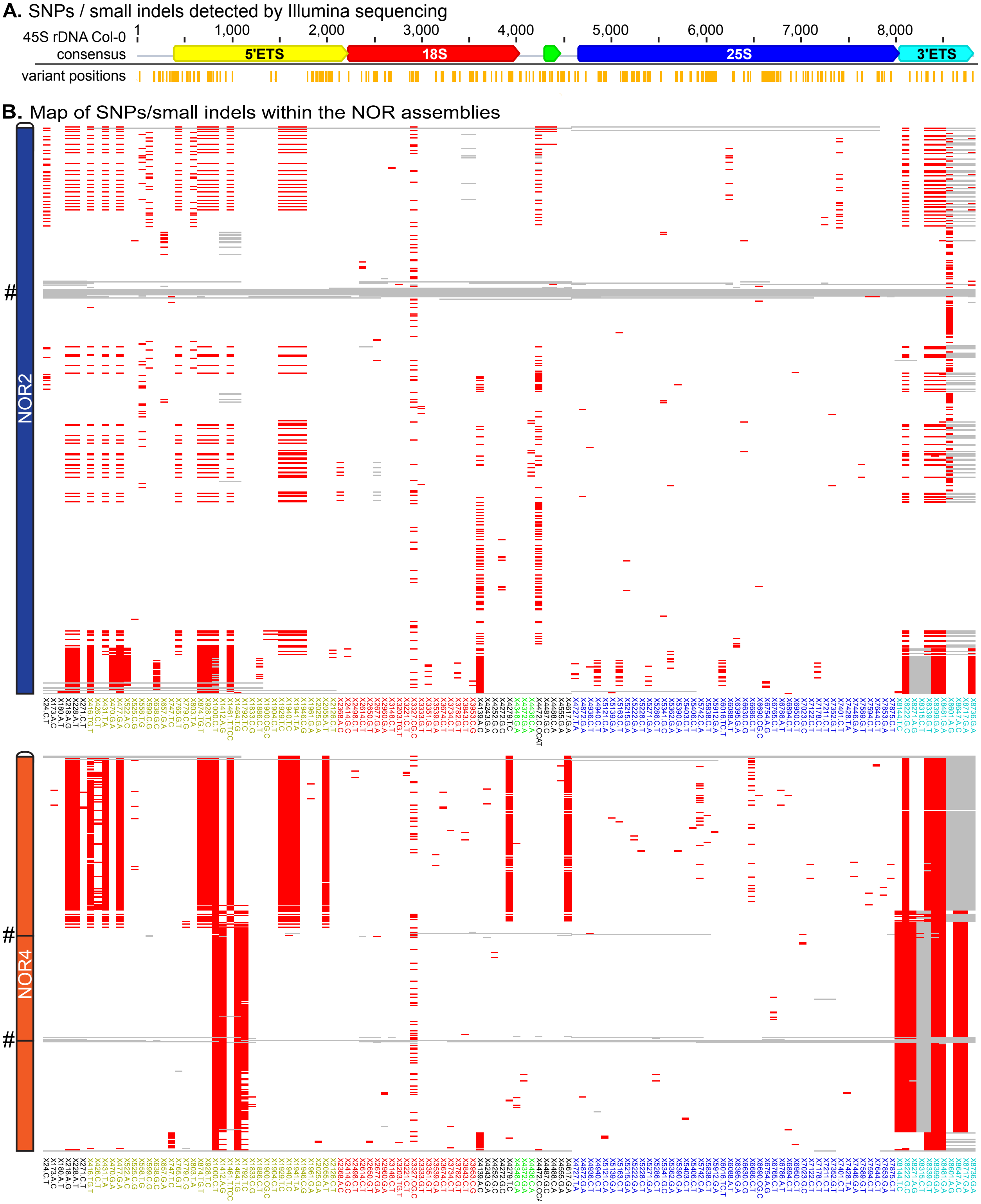

Fig. S6. Illumina sequencing-detected SNPs/small indel positions within individual genes of *NOR2* and *NOR4*.

(**A**) Vertical orange bars below the rRNA gene diagram represent positions of SNPs / small indels (relative to the reference gene) detected in Illumina sequencing reads using LoFreq. (**B**) Map of SNPs/small indels on the NOR assemblies. Each line on the Y axis represents an

individual 45S gene, from positions near the telomere (top) to centromere (bottom). Each column marks the presence (red) or absence (white) of SNPS/small indels. Gray represents no alignment to the consensus at that region. SNP labels on the x-axis are color-coded to match the regions in (A).

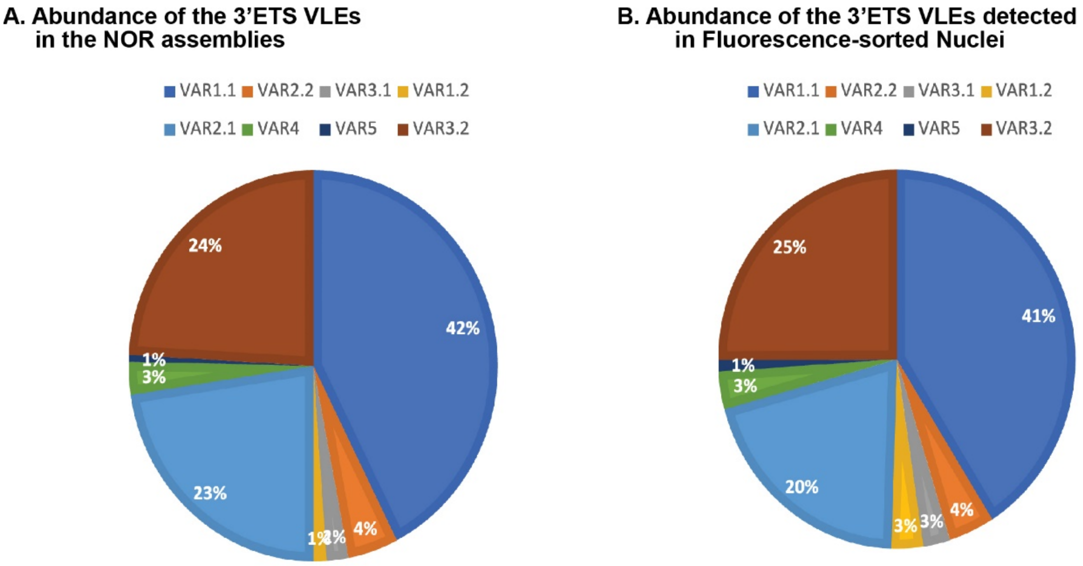

Fig. S7. rRNA gene subtype abundance in flow-sorted whole nuclei closely matches their abundance in the NOR sequence assemblies.

(**A**) Abundance of genes bearing the different 3'ETS VLEs in the NOR assemblies versus (**B**) the abundance of the genes bearing the different 3'ETS VLEs following their detection by ONT sequencing of DNA purified from flow-sorted nuclei. This experiment indicates that flow-sorting allows all subtypes to be detected without apparent bias.

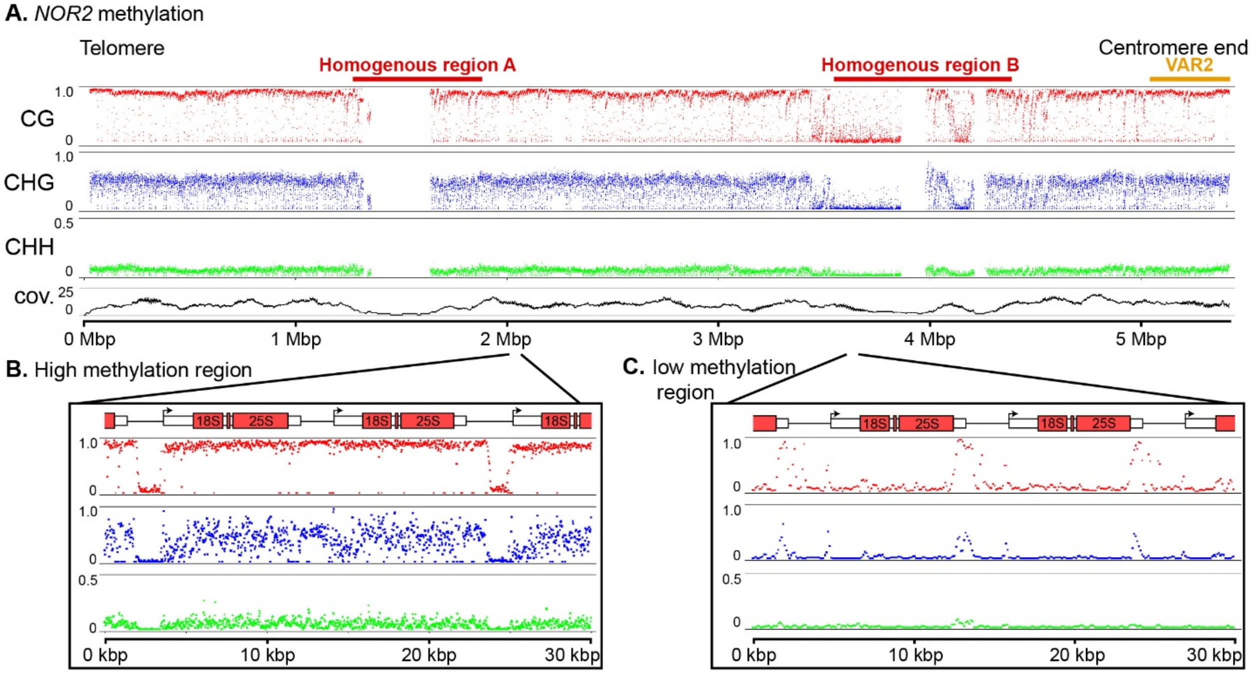

Fig. S8. Details of rRNA gene methylation patterns.

(**A**) 5mC frequencies in the CG, CHG, and CHH contexts at *NOR2* are shown, as in Figure 5A. (**B**) Zoomed-in view of a region characterized by high 5mC levels showing the dips in methylation that occur upstream of many, but not all, gene promoter regions. (**C**) Zoomed-in view of a representative region characterized by low 5mC levels, showing the characteristic spike in methylation observed at the 3'ETS region.

Table S1. ONT sequencing run statistics

Total output

ONT Sequencing Run	Run Name	# cells	total bp:		total bp per flowcell:	
			**100kb+**	**200kb+**	**100kb+**	**200kb+**
2022-05-12 Col-0	2022_5_12_CS1092_floapex_nuclei_ULK	2	6,020,711,635	1,561,915,198	3,010,355,818	780,957,599
2022-07-28 Col-0	2022_07_28_CS1092_infloapex_ULK	5	6,831,857,395	1,616,646,803	1,366,371,479	323,329,361
Combined runs		7	12,852,569,030	3,178,562,001		

45S gene output

ONT Sequencing Run	Run Name	# cells	45S rRNA gene total bp:		45S rRNA gene bp per flowcell:	
			**100kb+**	**200kb+**	**100kb+**	**200kb+**
2022-05-12 Col-0	2022_5_12_CS1092_floapex_nuclei_ULK	2	456,785,638	128,525,522	228,392,819	64,262,761
2022-07-28 Col-0	2022_07_28_CS1092_infloapex_ULK	5	581,480,077	157,498,739	116,296,015	31,499,748
Combined runs		7	1,038,265,715	286,024,261		

bp in quality-controlled assembly read set:
931,988,411

Table S2.

Sequencing data statistics for fluorescense-sorted nuclei and nucleoli

	Total reads	Total DNA bases	Read Length (N50)	45S ribosomal DNA reads	45S ribosomal DNA bases	45S ribosomal DNA Read Length (N50)
WT Nuclei	869,619	1,428,395,669	3,180	84,472	185,912,945 (13% of Total)	4,285
WT Nucleoli	77,876	113,550,068	2,615	33,713	57,184,319 (50% of Total)	2,864
hda6 Nuclei	527,843	874,477,467	3,569	23,204	73,745,623 (8.4% of Total)	6,797
hda6 Nucleoli	48,148	91,843,471	4,627	12,357	35,787,141 (39% of Total)	5,619

Data S1. (separate file)

45S rRNA gene consensus reference sequence (fasta file) used for VLE analyses and sequence for subtype #10, used for dot-plot analyses of ONT reads

Data S2. (separate file)

NOR assembly landmarks, observed vs. predicted coverage based on sequencing depth.

Data S3. (separate file)

ONT read alignments based on VLEs for *NOR2* telomere-proximal end.

Data S4. (separate file)

ONT read alignments based on VLEs for *NOR2* centromere-proximal end.

Data S5. (separate file)

ONT read alignments based on VLEs for *NOR4* telomere-proximal end.

Data S6. (separate file)

ONT read alignments based on VLEs for *NOR4* central region.

Data S7. (separate file)

ONT read alignments based on VLEs for *NOR4* centromere-proximal end.

Data S8. (separate file)

Nucleotide accuracy for NOR assemblies.

Data S9. (separate file)

NOR assemblies capture the VLE content of the sequencing reads.

Data S10. (separate file)

The NOR assemblies represent the variation detected in Illumina sequencing.
